# Deeper wedge resection and parenchymal-sparing bronchoplasty of the secondary carina: an alternative surgical technique for removal of tumor located at the orifice of upper lobar bronchus

**DOI:** 10.1186/s13019-017-0610-8

**Published:** 2017-06-13

**Authors:** An Wang, Xiaofeng Chen, Dayu Huang, Shaohua Wang

**Affiliations:** Department of Thoracic Surgery, Huashan Hospital, Fudan University, Shanghai, China

**Keywords:** Secondary carina reconstruction, Bronchoplasty, Parenchymal-preserving surgery

## Abstract

**Background:**

Sleeve resection and reconstruction of the bronchial corner between the upper lobar bronchus and the intermediate bronchus is technique demanding.

**Case presentation:**

A 33-year-old Chinese man suffered from recurrence of low-grade malignant mucoepidermoid carcinoma located at the orifice of upper lobar bronchus with invasion to the main bronchus nearly 1 year after he had undergone an incomplete bronchoscopic resection. With detailed preoperative and intraoperative evaluation by computed tomography and bronchoscopy, a deeper wedge resection and bronchoplasty of the secondary carina was performed. The freedom from tumor cells at the cut-edges was guaranteed by frozen examination. The postoperative course was uneventful and the patient was free from recurrence for 18 months after the surgery.

**Conclusions:**

With an R0 resection, the procedure described in the present case report was feasible and relatively easy, thus an alternative to sleeve lobectomy or sleeve bronchial resection for small-size low-grade malignancy located at the orifice of upper lobar bronchus.

## Backgroud

Sleeve resection and reconstruction of the bronchial corner between the upper lobar bronchus and the intermediate bronchus is technique demanding with acceptable mortality and morbidity due to improved surgical technique and postoperative care [1]. Here we reported a case of deeper wedge resection and bronchoplasty of the secondary carina for tumors located at the orifice of upper lobar bronchus with main bronchus involvement. The parenchymal-sparing surgical technique has not been detailed described in the literatures and was believed to be easier and less time-consuming than sleeve bronchial resection.

## Case presentation

**Fig. 1:**
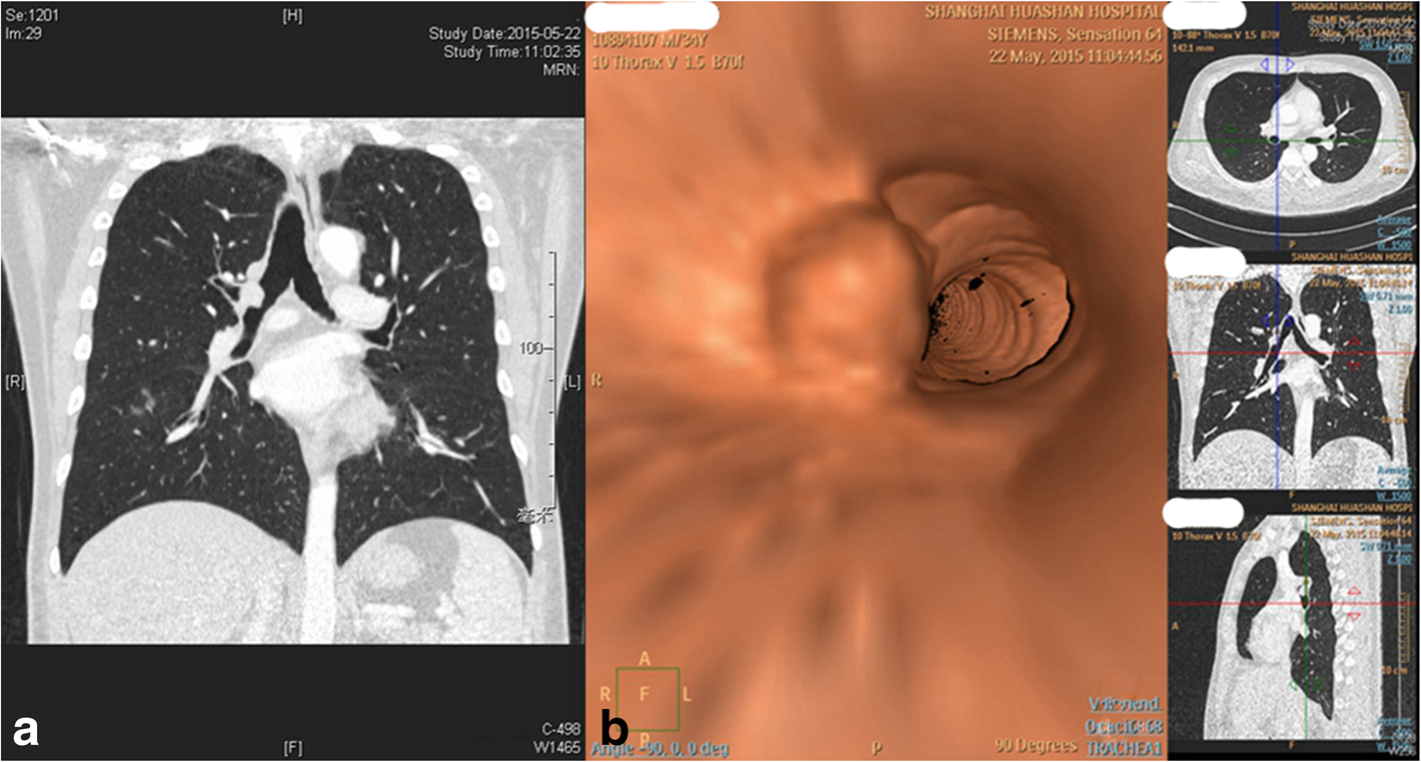
**a** Multi-planar reconstruction (MPR) by chest CT showing a mass approximately 1.3 cm in diameter at the onset of right upper lobar bronchus; **b** Virtual bronchoscopy showing a neoplasm protruding from the right upper lobar bronchus to the main bronchus

A 33-year old man was admitted with a complaint of exacerbated cough and hemoptysis for nearly one year since he underwent a bronchoscopic procedure for mucoepidermoid carcinoma resection at the orifice of right upper lobar bronchus in his local hospital. After computed tomography (CT) scanning, a mass of 13 mm in size situated at the junction of the right upper lobar bronchus and main bronchus was found (Fig. 1). Bronchoscopic biopsy confirmed the diagnosis of mucoepidermoid carcinoma recurrence.

**Fig. 2:**
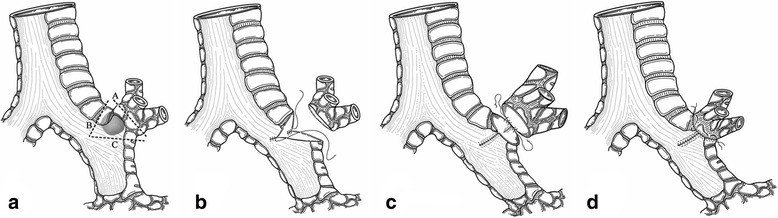
Schematic diagrams of the surgery. **a** According to the CT findings and intraoperative bronchoscopic guidance, three incision lines were scheduled for wedge resection of the right main bronchus where the upper lobar bronchus situated; **b** Deeper wedge resection of the bronchus. The incision edge was free of tumor, confirmed by frozen pathological examination; **c** 3–0 absorbable suture was used to partly suture the proximal and distal incision line, with a hole left and trimmed by scissors to match the orifice of the upper lobar bronchus; **d** 4–0 absorbable suture was used to perform an end-to-side anastomosis to restore the upper lobar bronchus and reconstruct the secondary carina

Surgical therapy was administered. The patient was placed in the left lateral decubitus position. General anesthesia was induced with a double lumen endotracheal tube in trachea. A 10 cm muscle-sparing incision was made in the fifth intercostal space. Firstly, hilar lymph nodes (#10), inter-lobar lymph nodes (#11 s) and the lymph nodes around the upper lobar bronchus (#12u) were removed, and the right main bronchus and the secondary carina were mobilized circumferentially. Then, an R0 deeper wedge resection of the main bronchus where the tumor located was made, with the secondary carina reconstructed and the upper lobe restored (Fig. 2). No air leak was found by water-seal test.

**Fig. 3:**
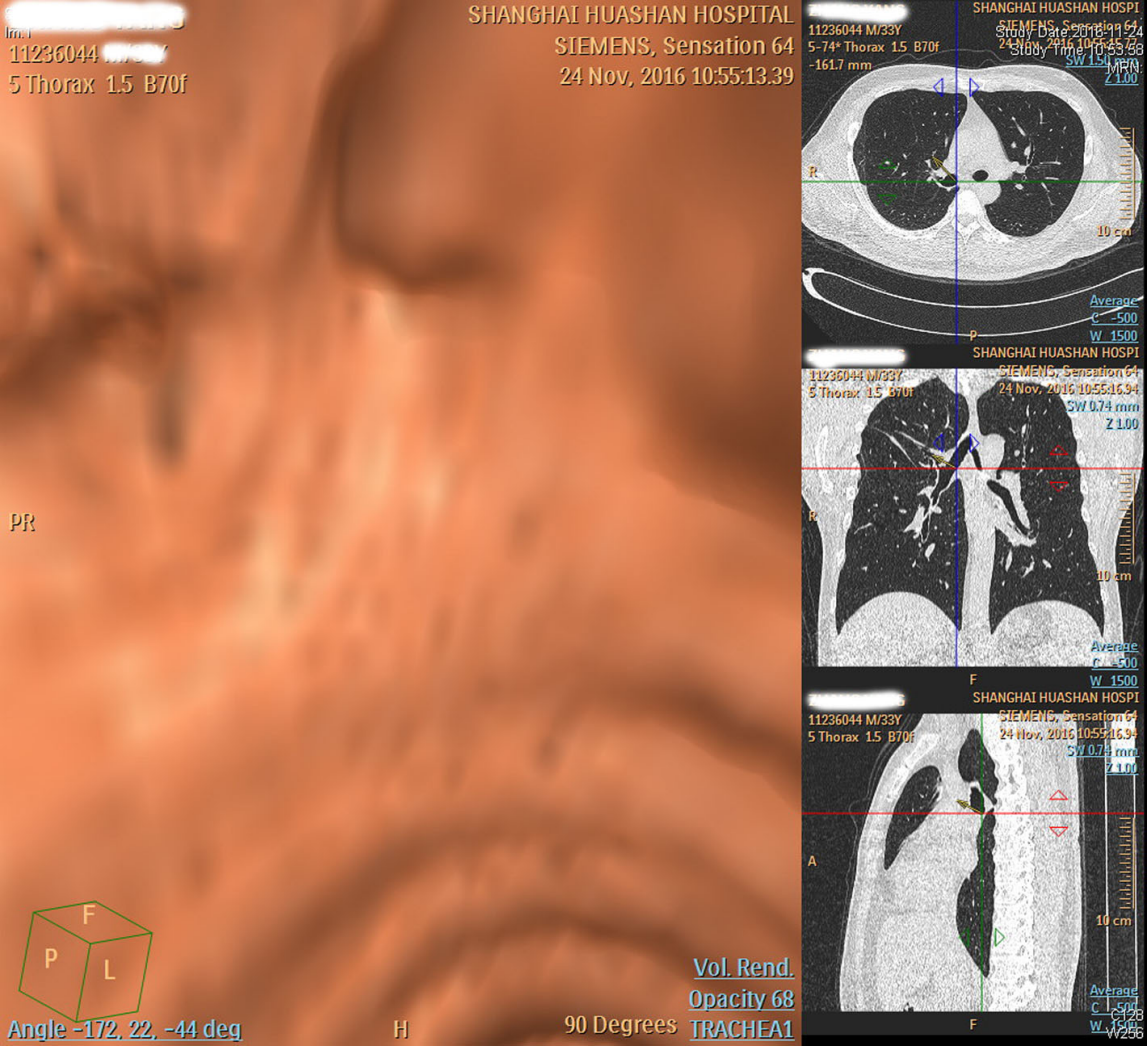
Postoperative virtual bronchoscopy by chest CT showing the anastomotic region where the tumor developed preoperatively and multi-planar reconstruction (MPR) by chest CT showing patency of the right main bronchus, intermediate bronchus and upper lobar bronchus without any evidence of recurrence and atelectasis

The pathological examination was compatible to the recurrence of mucoepidermoid carcinoma of low grade with no lymph node involvement. The postoperative course was uneventful. He remained free of tumor 18 months after surgery (Fig. 3).

## Discussion

Since the first report of bronchoplasty [2], a variety of surgical technical modifications has been reported, and with the development of surgical technique, the morbidity and mortality has been decreasing in these years to an acceptable level [3–5]. The surgical technique described here differed from the previous methods in the following aspects: (1), wedge but not sleeve resection was made at the secondary carina; (2), the upper lobe was divided and then restored by the secondary carina reconstruction with the tumor resected.

Compared to the sleeve resection of the right main bronchus, the present method was supposed to be easier, less time-consuming and less technique demanding, with more blood-supply preserved; Compared to sleeve lobectomy, the present method preserved pulmonary parenchymal; Compared to wedge resection of the bronchus without secondary carina reconstruction, in which too short proximal and distal incision line made angular deformity after suturing, the deeper wedge resection and subsequent reconstruction was free of such concern due to the orifice left in the lateral wall of the main bronchus for the restoration of the upper lobe.

The key points of the method were the incision lines of wedge resection. Firstly, the incision line of bronchotomy should be determined with intra-operative bronchoscopic guidance. The coronal scan could also play a role in determining the incision lines. Secondly, three incision edges should be checked respectively by frozen examination to ensure no involvement of tumor cell. In case of tumor cell involvement in incision line A or B (Fig. 2a), deeper wedge resection should be added or abandoned, substituted by sleeve bronchial resection. In case of tumor cell involvement in incision line C, further upper lobectomy should be considered.

## Conclusion

As a modification of surgical technique, our method is less technique-demanding and less time-consuming than previously reported method. It is eligible and thus an option for small-size low-grade neoplasms located at the orifice of upper lobar bronchus with invasion to main bronchus but intact median wall of main bronchus.

## Abbreviation

CT: Computed tomography
VATS: Video-assisted thoracoscopic surgery
